# Systematic analysis based on the cuproptosis-related genes identifies ferredoxin 1 as an immune regulator and therapeutic target for glioblastoma

**DOI:** 10.1186/s12885-023-11727-z

**Published:** 2023-12-19

**Authors:** Lirui Dai, Peizhi Zhou, Liang Lyu, Shu Jiang

**Affiliations:** Department of Neurosurgery, Pituitary Adenoma Multidisciplinary Center, West China Hospital of Sichuan University, Chengdu, 610041 Sichuan China

**Keywords:** Glioblastoma, Cuproptosis, Prognostic model, Drug, FDX1, Immune infiltration

## Abstract

Glioblastoma multiforme (GBM) is recognized as the prevailing malignant and aggressive primary brain tumor, characterized by an exceedingly unfavorable prognosis. Cuproptosis, a recently identified form of programmed cell death, exhibits a strong association with cancer progression, therapeutic response, and prognostic outcomes. However, the specific impact of cuproptosis on GBM remains uncertain. To address this knowledge gap, we obtained transcriptional and clinical data pertaining to GBM tissues and their corresponding normal samples from various datasets, including TCGA, CGGA, GEO, and GTEx. R software was utilized for the analysis of various statistical techniques, including survival analysis, cluster analysis, Cox regression, Lasso regression, gene enrichment analysis, drug sensitivity analysis, and immune microenvironment analysis. Multiple assays were conducted to investigate the expression of genes related to cuproptosis and their impact on the proliferation, invasion, and migration of glioblastoma multiforme (GBM) cells. The datasets were obtained and prognostic risk score models were constructed and validated using differentially expressed genes (DEGs) associated with cuproptosis. To enhance the practicality of these models, a nomogram was developed.Patients with glioblastoma multiforme (GBM) who were classified as high risk exhibited a more unfavorable prognosis and shorter overall survival compared to those in the low risk group. Additionally, we specifically chose FDX1 from the differentially expressed genes (DEGs) within the high risk group to assess its expression, prognostic value, biological functionality, drug responsiveness, and immune cell infiltration. The findings demonstrated that FDX1 was significantly upregulated and associated with a poorer prognosis in GBM. Furthermore, its elevated expression appeared to be linked to various metabolic processes and the susceptibility to chemotherapy drugs. Moreover, FDX1 was found to be involved in immune cell infiltration and exhibited correlations with multiple immunosuppressive genes, including TGFBR1 and PDCD1LG2. The aforementioned studies offer substantial assistance in informing the chemotherapy and immunotherapy approaches for GBM. In summary, these findings contribute to a deeper comprehension of cuproptosis and offer novel perspectives on the involvement of cuproptosis-related genes in GBM, thereby presenting a promising therapeutic strategy for GBM patients.

**Supplementary Information:**

The online version contains supplementary material available at 10.1186/s12885-023-11727-z.

## Introduction

Glioblastoma multiforme (GBM) is a highly aggressive brain cancer characterized by rapid metastasis and a grim prognosis, resulting in approximately 200,000 annual deaths worldwide [1]. Despite the current standard treatment approach involving surgical resection and chemoradiotherapy, patients with GBM face a significantly poor prognosis, with a 5-year survival rate below 6.9 [2, 3]. Consequently, there is an urgent need to develop a novel prognostic model to identify high-risk patients and discover biomarkers for GBM diagnosis. This will enable accurate treatment selection and effectively enhance therapeutic outcomes and overall prognosis for individuals with GBM.

Tsvetkov et al. proposed that cuprotosis is a form of cell death that is dependent on copper and differs from known mechanisms of cell death, relying on mitochondrial respiration. Through their studies, it was discovered that copper binds to the lipoylated component of the tricarboxylic acid (TCA) cycle in the mitochondrial respiratory chain, resulting in the aggregation of lipoylated proteins and loss of iron-sulfur cluster proteins. These effects ultimately lead to cell death [4]. The researchers have identified several genes involved in regulating cell death induced by Cu2 + , such as FDX1, which has the ability to convert Cu2 + to the more harmful Cu1 + , as well as PDH complexes (DLAT, PDHA1, and PDHB) and LIPT1, LIAS, and DLD, which are responsible for the lipoic acid pathway [4]. An increasing number of researchers are currently investigating the association between cuproptosis and human diseases, including cancers [5], with the aim of identifying potential therapeutic strategies that can enhance patient survival and prognosis by comprehending the role of cuproptosis in the development of various diseases [6].

In recent times, a significant number of scholars have contributed to the field of cancer research by developing prognostic models based on cuproptosis-related genes, specifically for hepatocellular carcinoma [7], head and neck squamous cell carcinoma [8], and prostate cancer [9]. These models exhibit a high level of accuracy in predicting various aspects of cancer patients' prognosis, including their response to chemotherapy and potential for immunotherapy. This study aims to systematically construct a prognostic model for glioblastoma (GBM) by incorporating 18 cuproptosis-related genes. The accuracy of the model is validated using multiple datasets, and a nomogram is devised to enhance the practicality and applicability of the model. It is understood that cuproptosis is controlled by the lipid acylation of proteins mediated by mitochondrial ferredoxin 1 (FDX1), which serves as a crucial regulator of Cu2 + transport leading to cellular demise. Consequently, we conducted a comprehensive examination of the impact of FDX1 on various aspects of glioblastoma multiforme's (GBM) tumor microenvironment (TME), metabolic pathways, immune micro-environment, and drug responsiveness. To validate the expression and biological role of FDX1 in GBM, we specifically chose the LN229 and U251 cell lines for further investigation.

In summary, this study presents a promising prognostic prediction and precise therapeutic framework for individuals diagnosed with GBM, along with a potential target for therapeutic interventions.

## Materials and methods

### Data collection, preprocessing and normalization

The RNA sequencing (RNA-seq) data of 174 GBM patients and 1323 normal tissues were acquired from The Cancer Genome Atlas (TCGA), (https://portal.gdc.cancer.gov) [10] and Genotype-Tissue Expression (GTEx), (https://www.gtexportal. org/ home/) [11] datasets. Then, we integrated the expression data from acquired data, and executed the normalization to remove batch effects. The downloaded data were log2-trans-formed and the “sva” package in R software (version 4.2.0) were employed to batch normalization the above data [12].

### Differentially expressed cuproptosis‑related genes identification and analysis

Differentially expressed genes (DEGs) were obtained from 174 GBM tissues and 1323 normal samples using the "DESeq2" package, with screening conditions of adjusted *P* < 0.05 and fold change > 1.2. The volcano plot and heatmap of DEGs were generated using Xiantao Academic tools (https://www.xiantaozi.com/) and OmicStudio tools (https://www.omicstudio.cn/tool), respectively. Protein–protein interactions (PPI) of these DEGs were investigated using the string (https://string-db.org/) website [13].

### GSCALite database

Single Nucleotide Variation (SNV) module of GSCALite database(http://bioinfo.life.hust.edu.cn/web/GSCALite/) [14] presents the SNV frequency and variant types of the gene set in selected cancer types. They collected SNV data from NCI Genomic Data Commons (https://gdc.cancer.gov/) [15], SNV percentage of each gene's coding region was calculate by: Num of Mutated Sample/Num of Cancer Sample. The SNV summary, oncoplot and survival were all based on this data. On Copy Number Variation module, the statistics of hetero-zygous and homozygous CNV of each cancer type are dis-played as pie chat for gene set, and Pearson correlation is performed between gene expression and CNV of each gene in each cancer to help to analyze the gene expression signifi-cantly affected by CNV.

### Gene enrichment analysis of these DEGs

R packages, including “ggplot2”, “org.Hs.eg.db”, “enrichplot”, “GOplot” and “clusterProfiler” were employed to study the gene enrichment of 18 DEGs with GO and KEGG. KEGG database focuses on the analysis of metabolic pathways in living organisms.

### Identification of prognostic genes

Data was acquired from the TCGA and GTEx datasets, followed by the implementation of univariate Cox regression analysis using the "survival" R package. Subsequently, LASSO-Cox regression was employed to mitigate gene collinearity and decrease the number of DEGx. Finally, multivariate Cox regression analysis was conducted.

### Establishment and validation of a risk score model on account of DEGs

The risk score was assessed on account of standardized GBM mRNA expression data in the training set. The formula: Risk score = ∑(Coefi × Exp). Coefi indicates the coefficient of DEGs in LASSO-Cox regression analysis, Exp indicates DEGs expression. Subsequently, the GBM patient samples were categorized into high and low-risk groups using the median risk score, and the overall survival rate (OS) of these two groups was analyzed using Kaplan Meier survival analysis.. Finally, the receiver operating characteristic (ROC) curves were built to evaluate the prognostic performance of this model. Furthermore, we used the GEO cohort to verify the above model.

### Establishment of the DEGs nomogram system

We utilized various R packages, namely "RMS," "Survival," and "Regplot," to construct a nomogram. Each GBM patient's age, gender, 1p/19q codeletion, IDH status, WHO grade, and primary therapy outcome were assigned corresponding points, and the cumulative points for each GBM patient were calculated. Subsequently, we estimated the 1-year, 3-year, and 5-year survival rates based on the cumulative points. The obtained results were graphically represented using calibration curves.

### Gene expression, localization and survival prognosis analysis

UALCAN website (https://ualcan.path.uab.edu/) [16] was utilized to analyze the mRNA expression of FDX1 based on TCGA database. Meanwhile, Gene Expression Omnibus (GEO) (https://www.ncbi.nlm.nih.gov/gds) dataset [17] was employed to prove FDX1 expression. And cBioPortal (http://www.cbioportal.org/) website [18] was employed to analyze the co-located of FDX1 in glioma cell line. RNA-seq expression profiles for glioma were downloaded from the TCGA. Log-rank test was employed to contrast differences in survival between these groups. ROC analysis was utilized to compare the predictive accuracy of FDX1, while log-rank tests and univariate Cox proportional hazards regression were employed to determine P values, hazard ratio (HR), and 95% confidence intervals (CI) for the Kaplan–Meier curves.

### Metabolic pathway analysis of FDX1 in GBM

The GBM RNAseq data was obtained from The Cancer Genome Atlas (TCGA), and the genes associated with the respective pathway were collected and utilized through the employment of the Gene Set Variation Analysis (GSVA) package. Spearman correlation was employed to investigate the relationship between the genes and pathway scores.

### Relationship between FDX1 and tumor immune cell infiltration and immunoregulation-associated genes

The GBM RNAseq data were gained from TCGA, Chinese Glioma Genome Atlas (CGGA) (http://www.cgga.org.cn/) [19] and GEO datasets. In order to ensure a robust assessment of immune scores, we utilized the immunedeconv package, which incorporates six state-of-the-art algorithms, namely TIMER, xCell, MCPcounter, CIBERSORT, EPIC, and quanTIseq.

The TISDB (http://cis.hku.hk/TISIDB/) [20] and GDC (https://gdc.cancer.gov/) [21] datasets were employed to obtain the TCGA, CGGA and GEO databases on GBM. The potential of FDX1 in the immunotherapy of GBM was assessed through an examination of the correlation between FDX1 and genes related to anti-gene presentation, immune inhibition, immune stimulation, chemokine production, and chemokine receptor expression. The coordinate axis represents various datasets and genes, while the colors indicate the strength of the relationship between FDX1 and the respective genes.

### Drug sensitivity analysis

The sensitive score of each molecular compound was assessed using R package “pRRophetic”, and PubChem (https:// pubchem.ncbi.nlm.nih.gov/) website [22] was used to visualize the 3D structure of different drugs.

### Cell culture and transfection

The U251 and LN229 human GBM cell lines were acquired from Shanghai Zhong Qiao Xin Zhou Biotechnology Co., Ltd and maintained in DMEM medium (Biosharp) supplemented with 10% fetal bovine serum (ExCell Bio) at a temperature of 37℃ and a CO2 concentration of 5%. The small interfering RNAs (siRNAs) was purchased from RiboBio, and it included three different FDX1 siRNA sequences (FDX1-1: GGACAATATGACTGTTCGA, FDX1-2: GTCACCTCATCTTTGAAGA, FDX1-3: GGTGAAACATTAACAACCA). The lipofectamine® 3000 was purchased from Thermo Fisher Scientific.

### Western blot

The cells were detached using cell scrapers and subsequently incubated on ice for a duration of thirty minutes. Following this, the cell lysates were subjected to boiling at a temperature of 100℃ for a period of 5–10 min. The total protein content was then separated by electrophoresis using SDS-PAGE and subsequently transferred onto PVDF membranes. The blots were cut prior to hybridisation with antibodies during blotting. Afterwards, it was incubated overnight at 4℃ with primary antibodies against FDX1 (Cat. no. #ab108257; 1:1000; abcam), β-tubulin (Cat. no. #M20005, 1:5000; abmart), Vimentin (Cat. no. T55134, 1:1000; abmart), E-cadherin (Cat. no. TA0131, 1:1000; abmart), N-cadherin (Cat. no. T55015; 1:1000; abmart). The PVDF membrane was washed 3times, each time 10 min with TBST-Tween 20, and then incubated for 1–2 h with goat anti-rabbit lgG secondary antibody and goat anti-mouse lgG secondary antibody (Affinity HRP; 1:5000). Finally, washed the PVDF membrane and visualized with chemiluminescence (Cat. no. 34577; Thermo Scientific™).

### Colony formation assay

LN229 and U251 cells were digested with trypsin 24 h after transfection, and 500 cells were added into the new six-well plate, respectively. After a duration of 14 days, paraformaldehyde was employed for cell fixation, and crystal violet was utilized for cell staining. Subsequently, the six-well plates were gently rinsed with PBS, and the cells were enumerated.

### Wound healing assay

24–48 h after transfection of cells in six-well plates, scratches were produced in the middle of each six-well plate using a 200μl pipette tip, cleaned with PBS, observed and photographed with an inverted microscope. It was then placed in the incubator for normal cultivation and taken out again 48 h later on the inverted microscope for photography. Migration distance = Scratch width observed at (0h – 48h).

### Transwell assay

With regard to migration assay, 20,000 cells were blended into serum-free medium and put into the upper layer of the well, and medium including 10%FBS was put into the lower layer of the well. Subsequently, the cells were incubated for a duration of 48 h. In the case of the invasion assay, following the aforementioned procedures, matrigel was applied to the upper chamber prior to proceeding with the subsequent steps. Following a 48-h incubation period, the cells were fixed, stained, and subsequently photographed for analytical purposes.

### Statistical analysis

GraphPad Prism 9, SPSS 26.0 and R software (version 4.2.0) were employed to data analysis and visualization. Normally distributed data were expressed as the mean ± standard deviation (SD). Kaplan–Meier analysis was employed to assess the GBM patients’ survival time. Student’s t test was employed for comparing FDX1 expression between two groups. *P* < 0.05 was viewed as statistically significant. *: *P* < 0.05, **: *P* < 0.01, ***: *P* < 0.001.

## Results

### Identification of differentially expressed cuproptosis‑related genes between the tumor and normal tissues

**Fig. 1:**
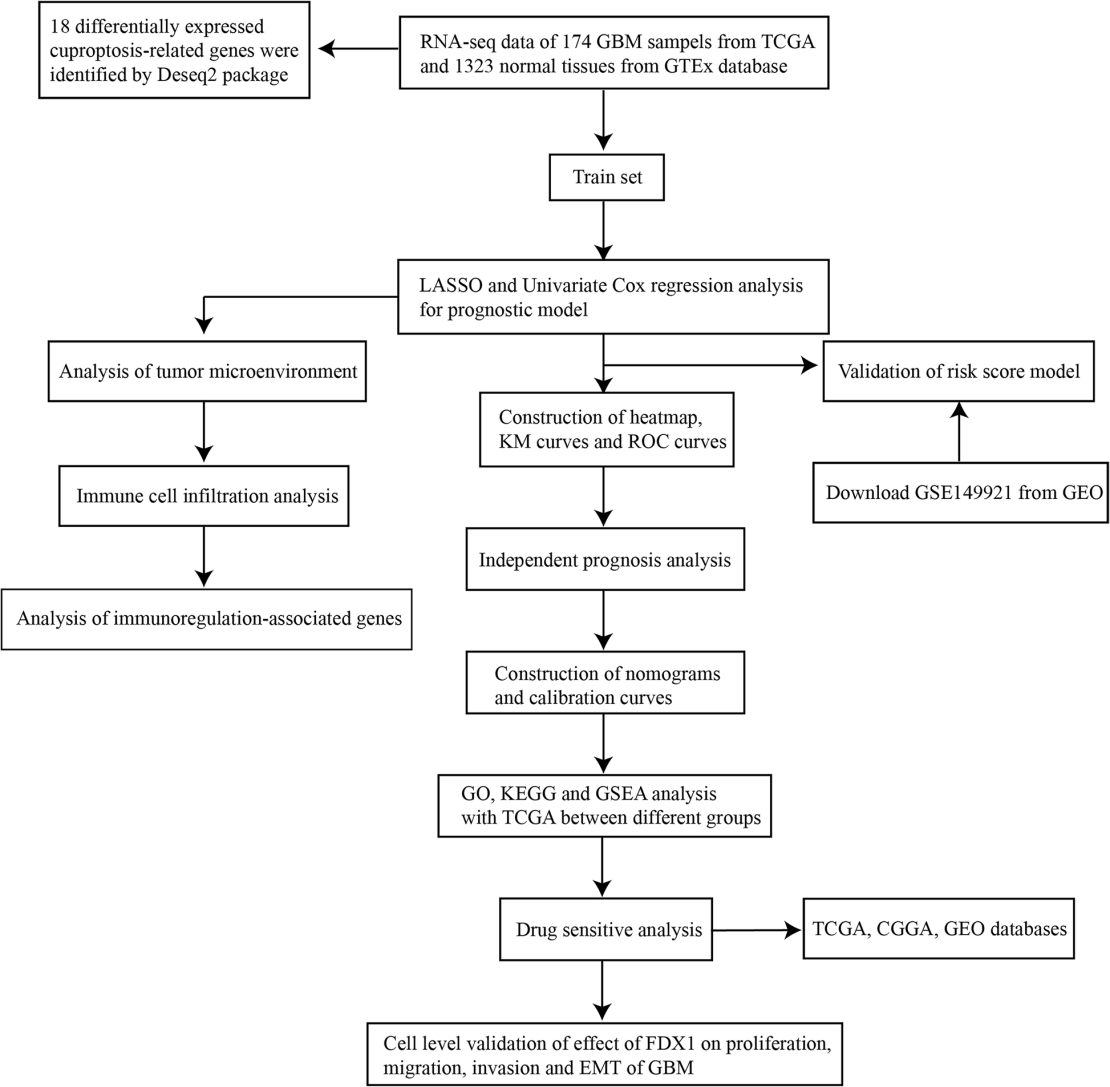
Workflow of the present study

**Fig. 2:**
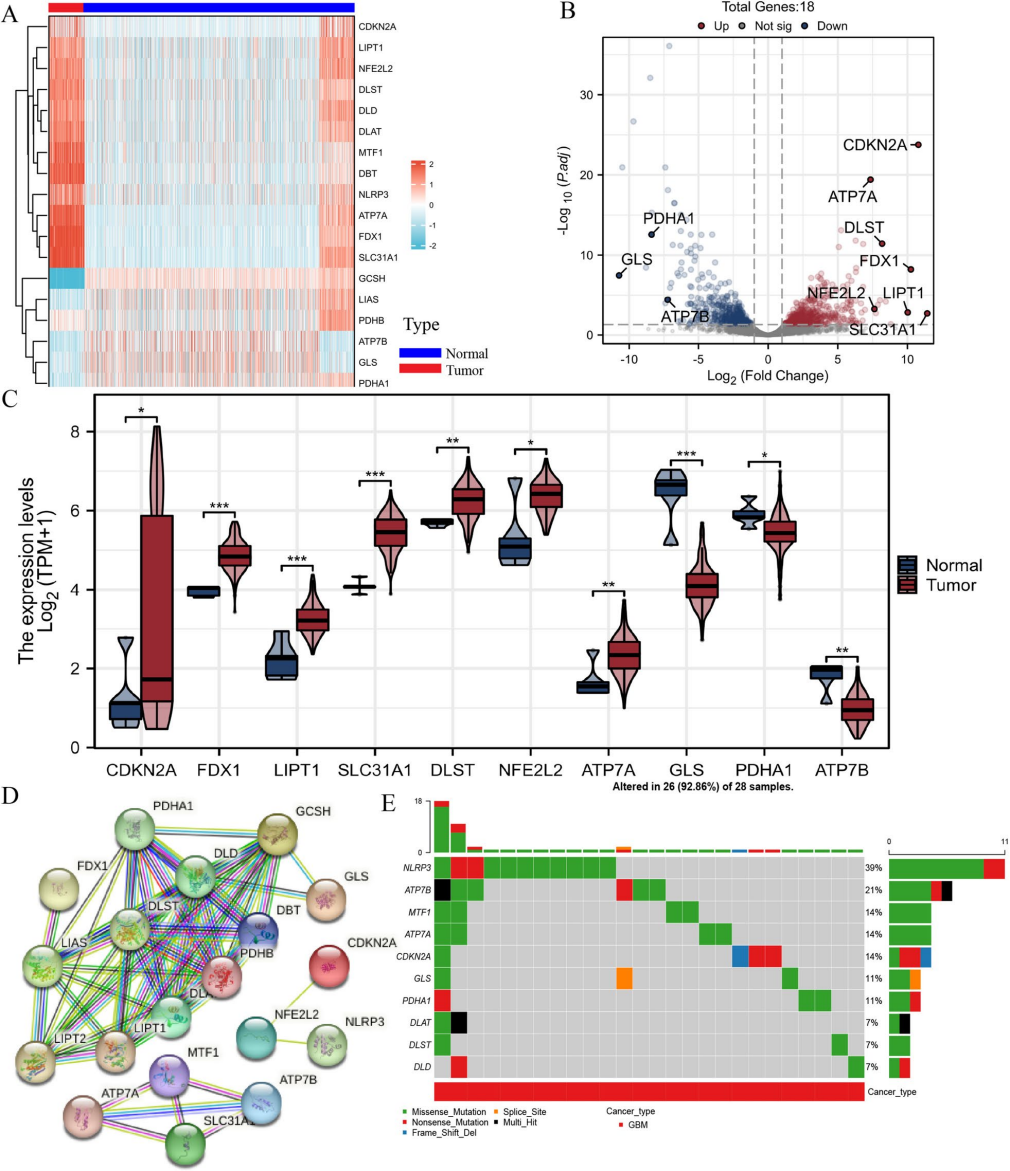
DEGs between GBM and normal samples. **A** Heatmap of DEGs. **B** Volcano plot showed cuproptosis-related genes.  **A** and **B**: Red: high expression, blue: low expression) **C** Boxplots of DEGs. Red: GBM samples, blue: normal samples. **D** The PPI network revealed the interaction of DEGs. **E** Mutation of DEGs in GBM patients.*: *P* < 0.05, **: *P* < 0.01, ***: *P* < 0.001

The flow chart designed in this paper is revealed in Fig. 1. The RNA-seq data of 174 GBM patients were acquired from TCGA and 1323 normal samples were obtained GTEx database. A total of 18 differentially expressed genes (DEGs) related to cuproptosis were identified based on the cutoff criteria of |log2 (fold change) |> 1.2 and false discovery rate < 0.05 using the "DESeq2" package [4, 23–25]. In GBM patients, we found that 12 cuproptosis-related genes were high expressed, while 6 cuproptosis-related genes were downregulated from the heatmap (Fig. 2A). Further examination through the use of a volcano plot (Fig. 2B) and boxplot (Fig. 2C) demonstrated that CDKN2A, FDX1, LIPT1, SLC31A1, DLST, NFE2L2, and ATP7A displayed significant upregulation, whereas GLS, PDHA1, and ATP7B exhibited marked downregulation in GBM.

The protein–protein interaction network in Fig. 2D shows the interaction of these cuproptosis-related genes. A summary analysis of simple nucleotide variations (SNV) in 28 GBM patients using GSCALite database showed that 26 (92.86%) patients had mutations in DEGs. Among these DEGs, NLRP3, ATP7B, MTF1, ATP7A, CDKN2A, GLS, and PDHA1 displayed a mutation frequency exceeding 10%, while DLAT, DLST, and DLD exhibited a mutation frequency below 10% (as shown in Fig. 2E). Subsequently, an examination of copy number variations (CNV) yielded a comprehensive analysis indicating widespread CNV loss in CDKN2A, ATP7B, DLST, PDHA1, ATP7A, DLAT, FDX1, GCSH, and LIAS, with CDKN2A exhibiting the most pronounced loss. reveals the interaction of these cuproptosis-related genes.Conversely, DLD, NLRP3, MTF1, DBT, SLC31A1, and PDHB displayed broad amplification, particularly DLD (Fig. S1). Nevertheless, the observed CNV alterations do not align consistently with the mRNA expression patterns observed in GBM and normal samples. This suggests that not only CNVs impacting the expression of differentially expressed genes (DEGs), but also other factors such as single nucleotide variations (SNV) and DNA methylation, may exert influence on the expression of DEGs.

### Functional enrichment analysis

Functional enrichment analysis was performed to better comprehend the biology functions of DEGs in GBM. The consequences of GO enrichment displayed that these DEGs were markedly correlated with the adjustment of mitochondrial matrix, cellular copper ion homeostasis and transition metal ion transmembrane transporter activity (Fig. S2A). Furthermore, the KEGG analysis revealed a strong correlation between the differentially expressed genes (DEGs) and the regulation of platinum drug resistance, central carbon metabolism in tumors, citrate cycle (TCA cycle), mineral absorption, and carbon metabolism (Fig. S2B). This observation implies a significant association between these DEGs and the regulation of cellular metabolism, biological functions, tumor regulation, and therapy.

### Establishment and validation of a prognostic model on account of DEGs between the sample subgroups

**Fig. 3:**
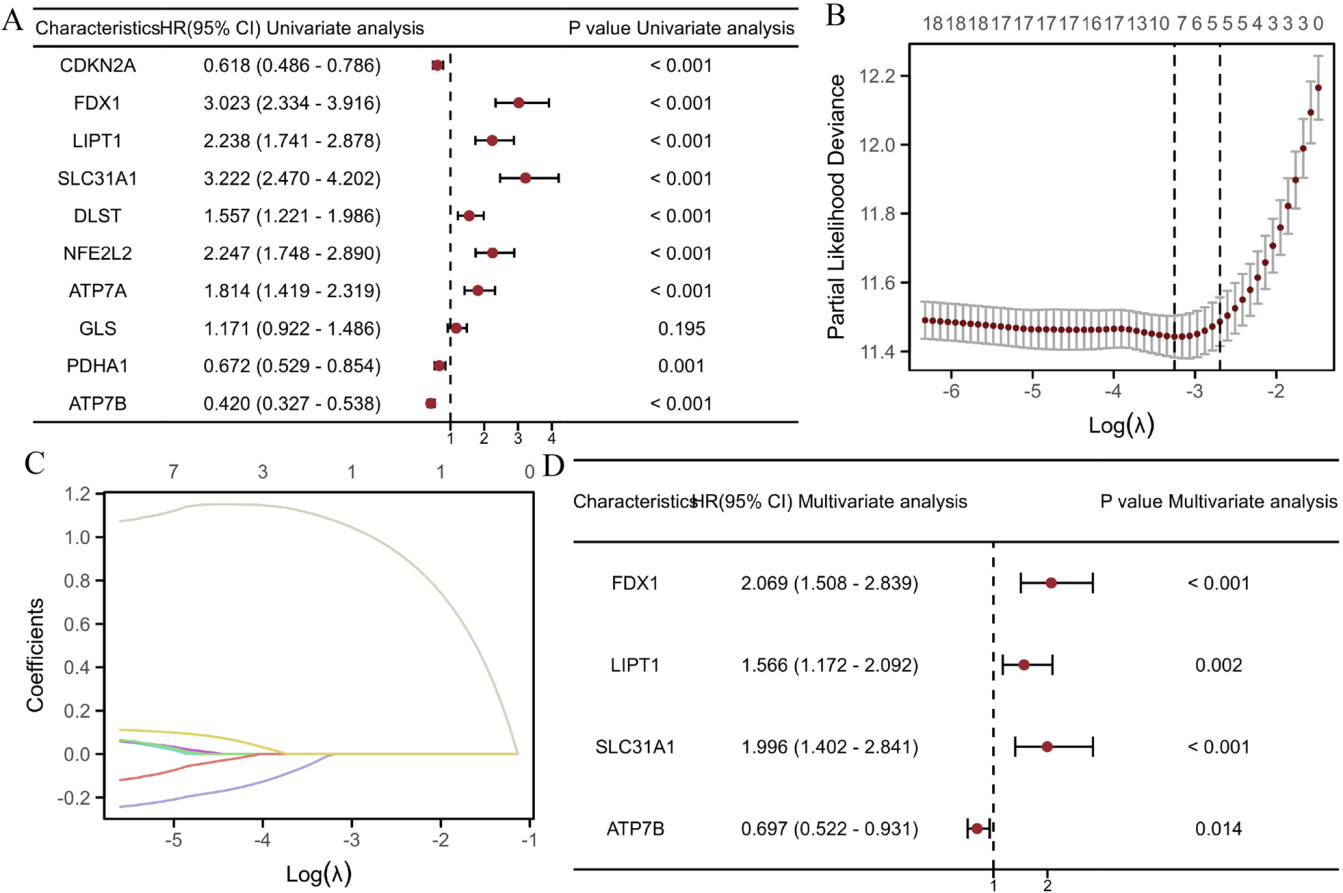
Establishment of a risk prognostic model on account of DEGs. **A** Univariate Cox regression analysis based on DEGs. **B** LASSO regression of the OS-related genes. **C** Cross-validation of adjustment parameter selection in LASSO regression. **D** Multivariate Cox regression analysis

The study utilized univariate Cox regression analysis to identify nine cuproptosis-related genes that exhibited statistical significance. Among these genes, six were identified as potential risk factors (FDX1, LIPT1, SLC31A1, DLST, NFE2L2, and ATP7A), while three were identified as potential protective factors (CDKN2A, PDHA1, and ATP7B) (Fig. 3A). Subsequently, LASSO regression was employed to further refine the selection of predictive genes, leading to the development of a prognostic model for cuproptosis (Fig. 3B-C). Finally, we employed multivariate Cox regression analysis to identify four genes correlated with cuproptosis, including 3 potential risk genes (FDX1, LIPT1 and SLC31A1) and 1 potential protective gene (ATP7B) (Fig. 3D).

**Fig. 4:**
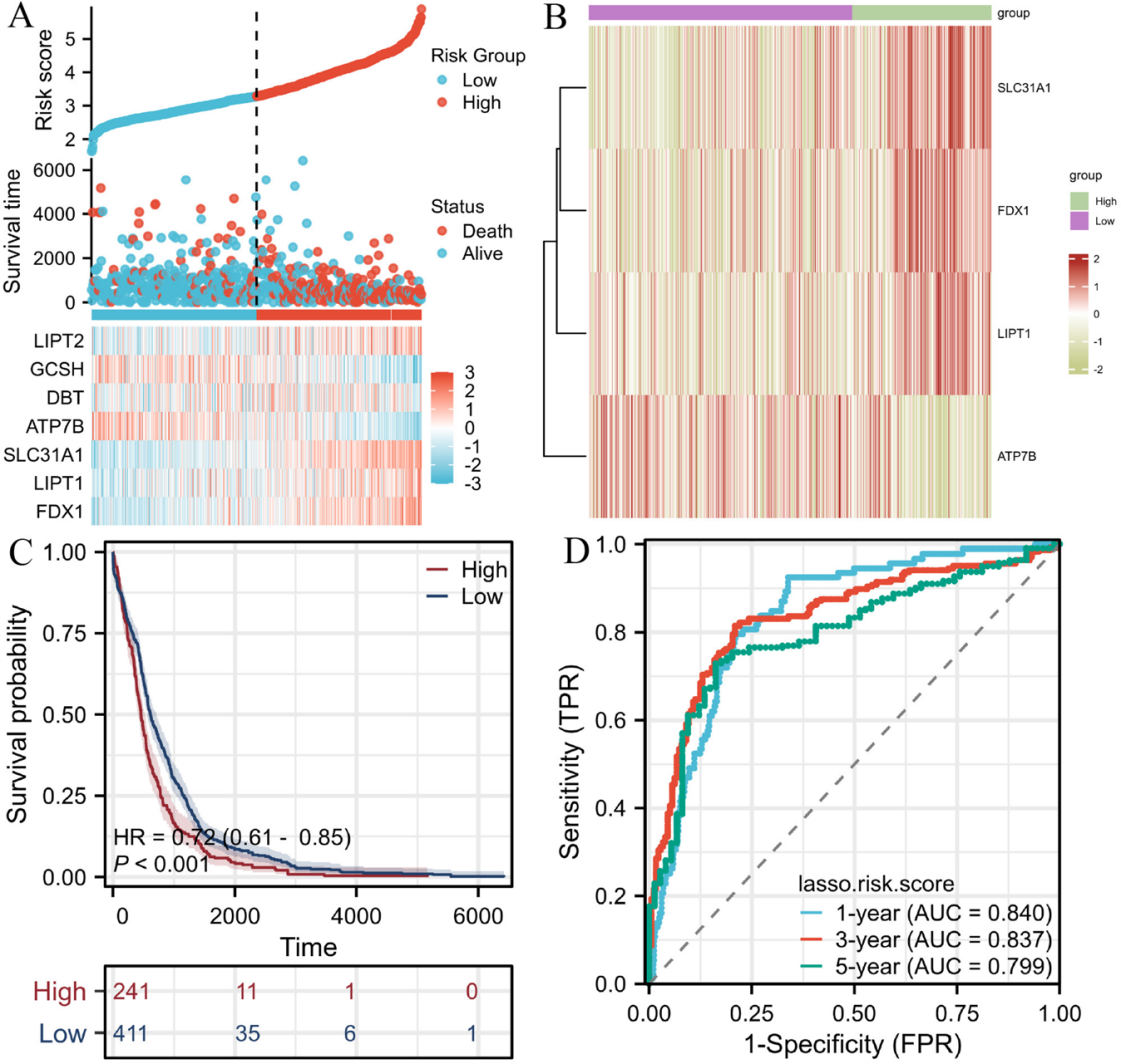
Establishment of risk model in TCGA dateset. **A** Patients were evenly divided into two groups on account of a threshold for the median risk score. Red: high-risk groups, yellow: low-risk groups. **B** Heatmap revealed four DEGs expression. Purple: low-risk groups, green: high-risk groups. **C** The Kaplan–Meier curve reveals the OS of patients in the high and low risk groups. **D** The ROC curve reveals the predictive efficiency of the risk score

A prognostic risk model was established using LASSO Cox regression analysis to identify high and low risk genes, namely FDX1, SLC31A1, LIPT1, and ATP7B. The risk score formula was derived as follows: risk score = 0.374273 × FDX1 expression + 0.211523 × LIPT1 expression + 0.671719 × SLC31A1 expression + (- 0.768527) × ATP7B expression. To investigate the predictive ability of this cuproptosis-related model in GBM patients, a total of 652 patients were selected based on the median risk score threshold and divided into high risk (*n* = 241) and low risk (*n *= 411) groups. According to the results (Fig. 4A), the low-risk group had a lower mortality rate and lived longer. FDX1, SLC31A1 and LIPT1 were significantly highly expressed in the high risk group, while ATP7B expression was reversed (Fig. 4B). A Kaplan Meier curve revealed that patients at low risk had a better prognosis, while those at high risk had a worse prognosis (Fig. 4C). Subsequently, we performed a time-dependent ROC analysis, which revealed that the prognostic accuracy of OS was 0.840, 0.837 and 0.799 at 1- year, 3- year and 5- year (Fig. 4D). These findings suggest that the cuproptosis-related model we developed has the potential to serve as a valuable tool for predicting the prognosis of patients with glioblastoma multiforme (GBM), exhibiting a commendable level of accuracy.

In order to validate the reliability of our prognostic model for cuproptosis, we obtained glioma patient data from the GSE149921 database and utilized the aforementioned formula to evaluate the risk score. Based on the median risk score, glioma patients were categorized into high-risk and low-risk groups. The findings of our analysis were consistent with the results, as FDX1, SLC31A1, and LIPT1 exhibited elevated expression levels in the high-risk group, whereas ATP7B displayed a reversed pattern. Furthermore, patients in the low-risk group demonstrated prolonged survival duration. Time dependent ROC analysis revealed that the prognostic accuracy of OS was 0.811, 0.805 and 0.776 at 1- year, 3- year and 5- year (Fig. S3). The above results show that the model has high accuracy in predicting GBM patients’ prognosis.

### Establishment of nomogram and calibration curves to predict survival

**Fig. 5:**
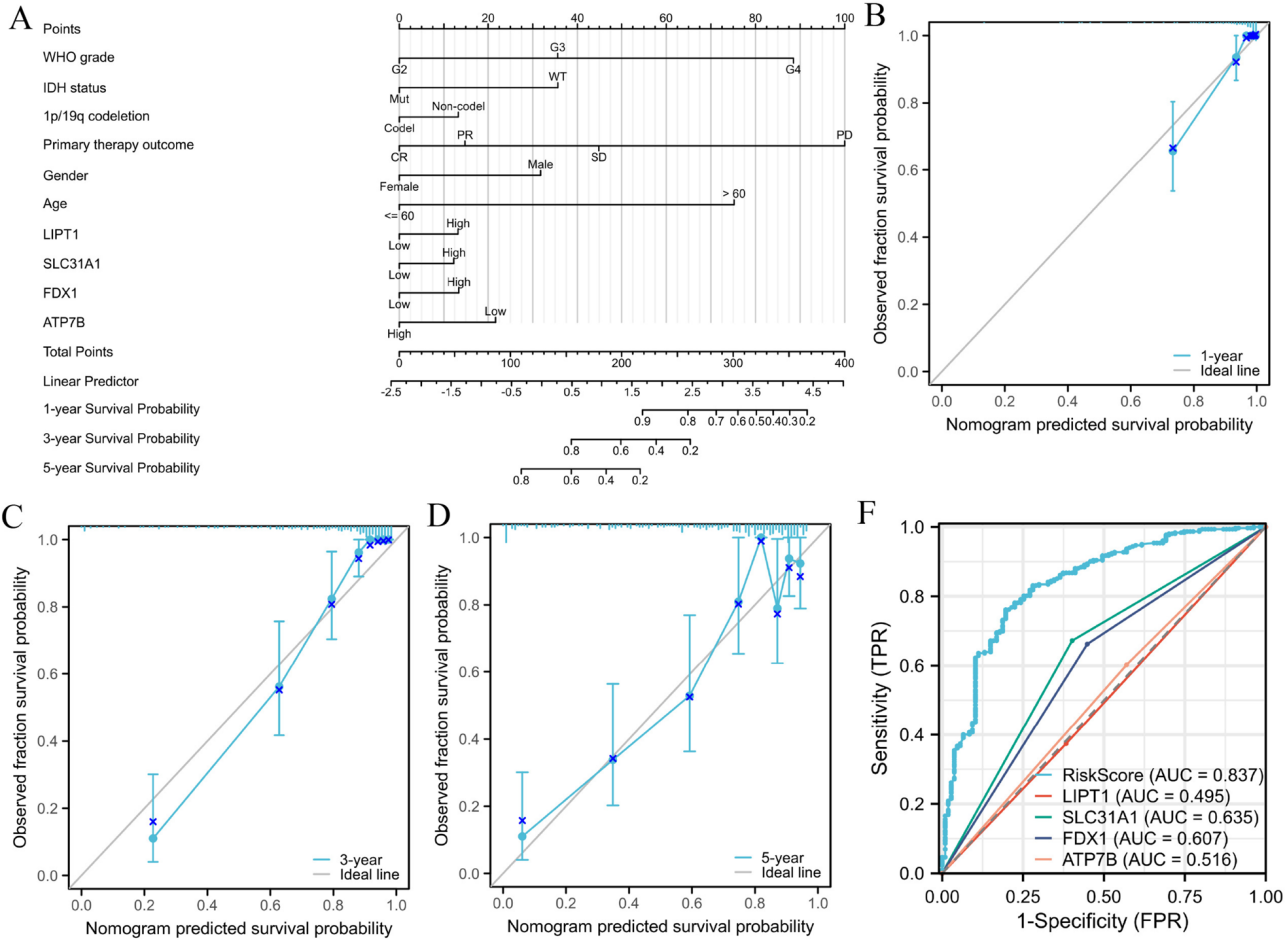
Nomogram to predict survival probability of GBM patients. **A** Nomogram combining risk score with WHO grade, IDH status, 1p/19q codeletion, gender, age and primary therapy outcome. **B-D** Calibration plots for predicting 1-, 3-, and 5-year OS of GBM patients. **E** The combining of 1-, 3-, and 5-year OS of GBM patients. **F** The ROC curves reveal the survival using the risk score

For purpose of increasing the clinical applicability of the model, we produced a nomogram including age, sex, primary treatment outcome, 1p/19q co-deletion, and WHO grade (Fig. 5A). These predictors were conducted to predict the 1-, 3- and 5-year survival rate of GBM patients. For example, a male GBM patient would receive 32 points for being male, 75 points for being over 60 years old, 45 points for having a stable disease (SD) as the primary therapy outcome, 13 points for not having a 1p/19q co-deletion, 35 points for having an IDH status of wild type (WT), and 88 points for having a WHO grade of G4. The cumulative score of 288 indicates that the 1-year survival rate for GBM patients is 65%, while the 3- and 5-year survival rates are below 20%. Furthermore, the findings reveal that the primary therapy outcome has the greatest impact on both the overall score and the survival of patients in the multiple regression models. Additionally, the calibration curves we developed provide further evidence that the nomogram accurately aligns with the survival rate of GBM patients (Fig. 5B-D). Moreover, our model exhibits a higher AUC value (AUC = 0.837) (Fig. 5E). Overall, the nomogram we have constructed demonstrates accurate prediction of the overall survival of GBM patients.

### Ferredoxin 1 expression, localization and prognostic significance in GBM

**Fig. 6:**
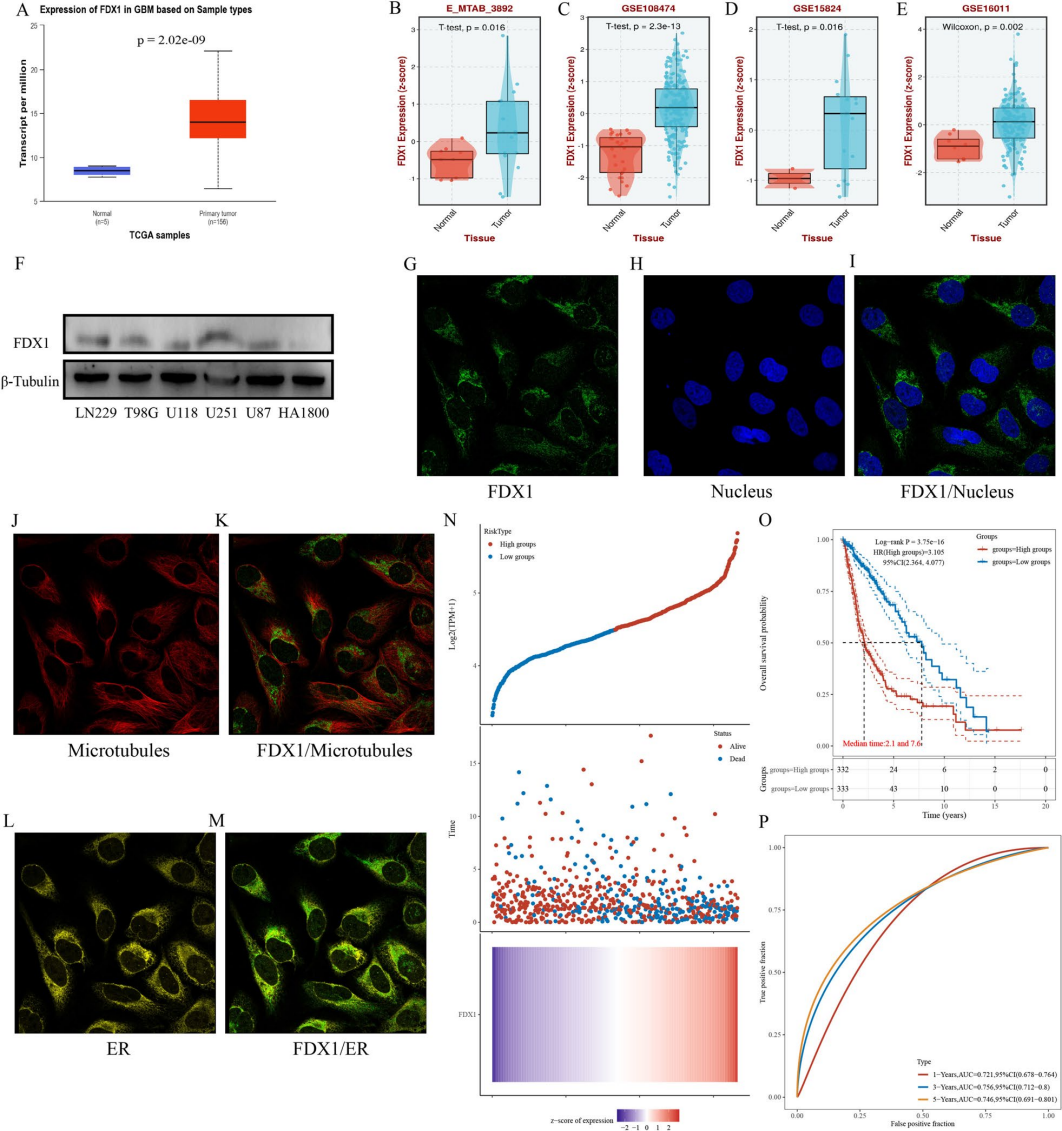
Expression, localization and prognostic value of FDX1 in GBM. **A** FDX1 expression in GBM on account of TCGA database. **B-E** FDX1 expression in GBM based on GEO and E_MTAB databases. **F** The protein expression of FDX1 in different GBM cell lines. **G-M** The localization of FDX1 in U251 cell line. **N** Survival time of GBM patients in high and low FDX1 expression groups. **O** The KM survival curve shows the OS of different groups. **P** The ROC curve was used to show the AUC scores of FDX1 at 1-, 3-, and 5-years

To further analyze the function and role of potentially risk genes in GBM, we selected FDX1 as the research object. TCGA database was used to compare FDX1 expression between GBM patients and normal samples. The result displayed that FDX1 expression was upregulated in GBM samples (Fig. 6A). For the purpose of proving the above results, we selected the E_MTAB_3892, GSE108474, GSE15824 and GSE16011 datasets for further analysis, and the consequences displayed that FDX1 was highly expressed in GBM (Fig. 6B-E). Additionally, a western blot analysis was conducted on five glioblastoma multiforme (GBM) cell lines (LN229, T98G, U118, U251, and U87) and one normal astrocyte (HA1800). The findings demonstrated a significant elevation in FDX1 protein expression within the five GBM cell lines compared to the normal control group (Fig. 6F). In conclusion, the results indicate an upregulation of FDX1 expression in GBM.

Subsequently, we employed the "Cell Atlas" segment of The Human Protein Atlas website to evaluate the localization of FDX1 in the GBM cell line (U251) via immunofluorescence analysis. The findings revealed that FDX1 exhibited co-localization with microtubules and endoplasmic reticulum (ER) markers in U251, with no discernible presence in the nucleus (Fig. 6G-M). These outcomes suggest a potential association between FDX1 and various cellular processes such as cell division, organelle transport, as well as carbohydrate and lipid synthesis and metabolism in GBM cells [26, 27].

Following this, we conducted a survival analysis of FDX1 in glioma. Utilizing TCGA data, we investigated the association between FDX1 expression and both survival time and survival status. The findings indicated that glioma patients with elevated FDX1 expression exhibited a higher mortality rate, while those with lower FDX1 expression demonstrated a higher survival rate (Fig. 6N). Additionally, Fig. 6O presented the Kaplan–Meier survival curve for FDX1 in TCGA, revealing a significantly lower overall survival rate in the high expression group compared to the low expression group..Finally, ROC curve was used, and find that the AUC values of FDX1 at 1- year, 3- year and 5- year are 0.721, 0.756 and 0.746. It is worth noting that a higher AUC value indicates a stronger predictive capacity of FDX1 (Fig. 6P). Consequently, FDX1 exhibits a significant potential for prognostic prediction in glioma patients, with higher expressionlevels correlating with poorer patient outcomes.

### GO, KEGG analysis and GSEA of FDX1 gene co-expression network in GBM

**Fig. 7:**
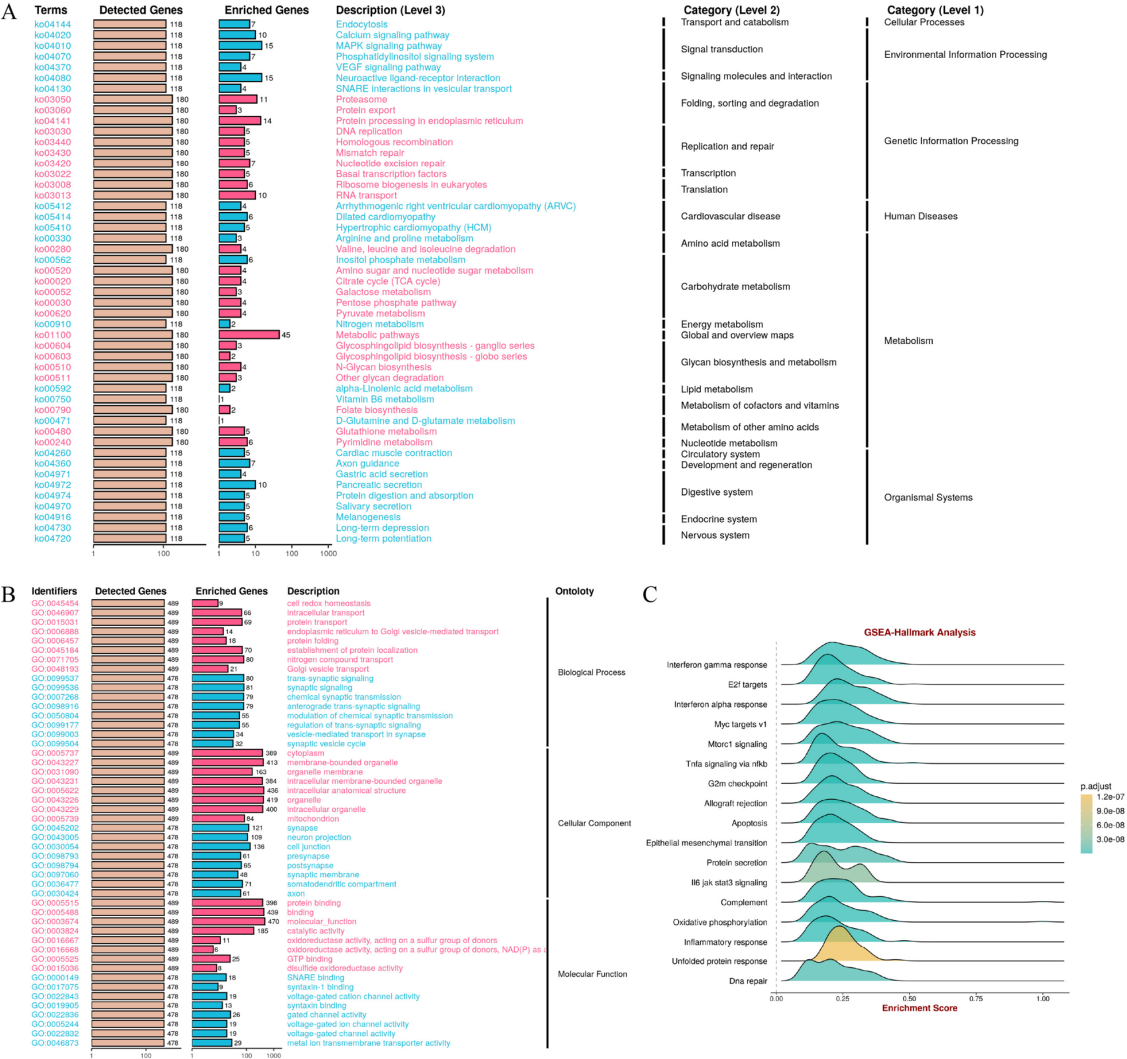
Go and KEGG pathways co-expression analysis and GSEA. **A** Go and **B** KEGG pathways co-expression of 500 FDX1-related genes in GBM RNA-seq data were analyzed in 2277 samples from 22 datasets. **C** GSEA analysis is used to further analyze the potential capabilities of FDX1

Go and KEGG pathways co-expression analysis of FDX1-related genes in GBM RNA-seq data with 2277 samples from 22 datasets were performed using the enrichment analysis module of “BEST” (https://rookieutopia.com/app_direct/BEST/). The top 500 related genes were analyzed, q-value cutoff: 0.05, GO-plot width: 0.4, KEGG-plot width: 0.4. The consequences of GO analysis displayed that these DEGs were relevant to various metabolism pathways, environmental information processing and genetic information processing (Fig. 7A). The KEGG analysis revealed that DEGs were associated with various molecular functions, including protein binding and catalytic activity. Additionally, these DEGs were found to be related to specific cellular components, such as membrane-bounded organelles, intracellular anatomical structures, and intracellular membrane-bounded organelles (Fig. 7B).

In order to conduct a more comprehensive examination of the potential roles of FDX1, gene set enrichment analysis (GSEA) was conducted on the DEGs. The results revealed significant associations between FDX1 and various immune-related pathways as well as metabolic responses in GBM, such as interferon gamma response, interferon alpha response, allograft rejection, and oxidative phosphorylation. Furthermore, FDX1 was found to play a role in the regulation of several biological functions, including epithelial mesenchymal transition, apoptosis, G2M checkpoint, and DNA repair (Fig. 7C).

### Metabolic pathway analysis of FDX1 in GBM

**Fig. 8:**
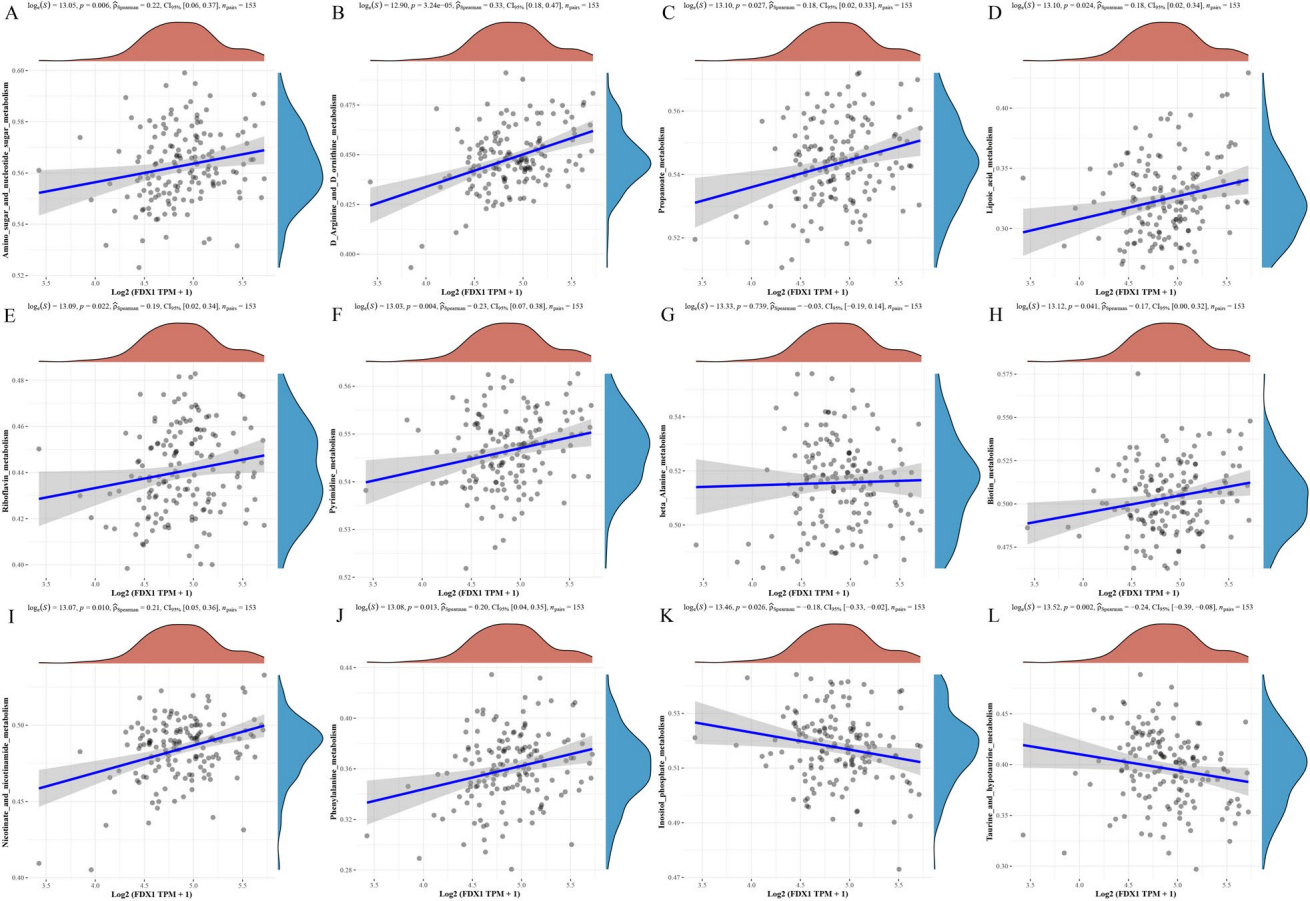
Metabolic pathway analysis of FDX1 in GBM. By collecting FDX1-related genes in metabolism-related pathways and calculating the enrichment score of each sample on each pathway on account of ssGSEA algorithm, the correlation between FDX1 and these pathways was obtained. **A-J** Metabolic pathways that are positively correlated with FDX1. **K-L** Metabolic pathways that are negatively correlated with FDX1

Based on the aforementioned enrichment analysis, it was observed that FDX1 exhibited significant relevance to numerous metabolic pathways. To establish the association between FDX1 and these pathways, we compiled the set of FDX1-related genes present in metabolism-related pathways and evaluated the enrichment score of each sample using the ssGSEA algorithm. The results displayed that FDX1 expression was positively correlation with multiple metabolic pathways, such as amino sugar and nucleotide sugar metabolism, propanoate metabolism, lipoic acid metabolism, riboflavin metabolism, nicotinate and nicotinamide metabolism and phenylalanine metabolism, while negatively associated with inositol phosphate metabolism and taurine and hypotaurine metabolism (Fig. 8). Of course, high FDX1 expression is also involved in many other metabolic and biosynthetic processes, such as biosynthesis of unsaturated fatty acids and cellular response to hypoxia (Figure S4). These findings strongly indicate that heightened FDX1 expression in GBM exerts regulatory influence on tumor progression through modulation of diverse metabolic processes. Consequently, this implies the potential for future therapeutic interventions in GBM by targeting metabolic pathways.

### Exploration of tumor microenvironment, immune cell infiltration and immunoregulation-related genes

**Fig. 9:**
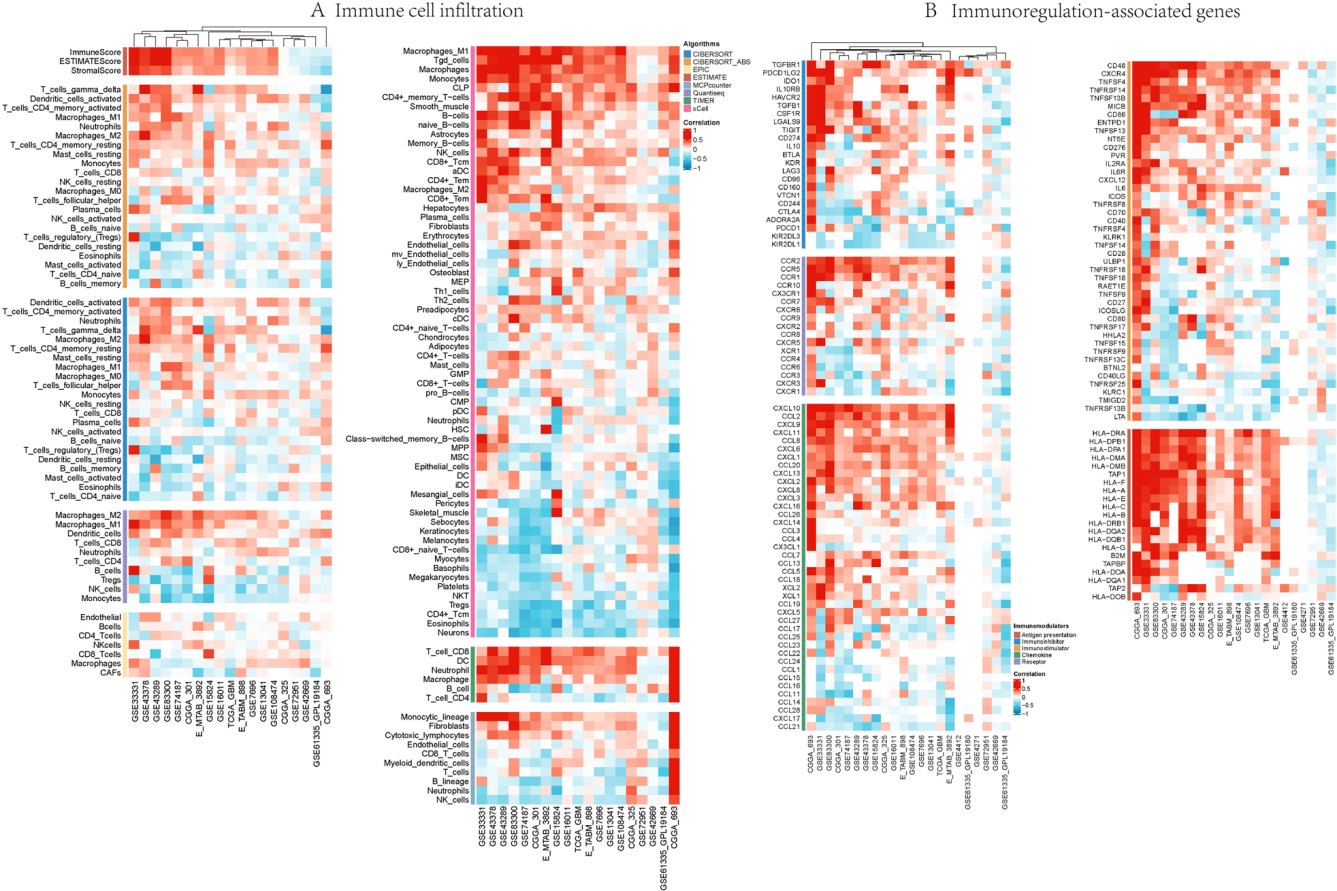
The relationship between FDX1 expression and immune cell infiltration and immunoregulation-associated genes. **A** The relationship of FDX1 and immune cell infiltration levels based on 7 different algorithms. **B** The correlation between FDX1 expression and immune-activating genes, immunosuppression-associated genes, chemokine receptors and chemokines based on TCGA, CGGA and GEO datasets

Since immune cell infiltration are significantly relevant to the occurrence and development of tumors, the proportion of immune cells and stromal cells in the TME has a significant effect on the prognosis, and is of great value for the diagnosis and prognosis assessment of the tumor. Based on TCGA, CGGA and 15 GEO datasets, we used multiple immune infiltration score algorithms to assess the immune score and matrix score, which mainly included three scores, including stromal, immune and estimate scores. The difference analysis showed that the distribution of immune cell infiltration between two groups. CIBERSORT algorithms displayed that FDX1 expression was observably relevant to Dendritic cells activated, T cells CD4 memory activated, Neutrophils, Macrophages M1 and Macrophages M2, while negatively correlation with B cells memory, T cells CD4 naive and Mast cells activated. In addition, quanTIseq algorithms significantly revealed that FDX1 expression was relevant to Macrophages cells, Tgd cells, Monocytes, CD4 + memory T cells and Smooth muscle, while negatively related to Neurons, Eosinophils, CD4 + Tcm, Tregs, NKT, Platelets, Megakaryocytes, Basophils and CD8 + naïve T cells. Other algorithms like EPIC, ESTIMATE, MCPcounter, TIMER and xCELL were also employed to compare the relationship between FDX1 expression and immune cells (Fig. 9A). The aforementioned studies provide evidence supporting the notion that the expression of FDX1 can exert an influence on the immune activity within the tumor microenvironment (TME) in GBM.

The correlations between immune checkpoint genes, anti-gene presentation related genes, immune inhibited genes, immune stimulated genes, chemokine and chemokine receptor genes were evaluated in light of their significance for tumor immunotherapy. The findings revealed a positive association between FDX1 expression in GBM and various immune stimulated genes, including CD48, CXCR4, TNFSF4, TNFRSF14, TNFSF13B, and MICB. Conversely, FDX1 expression showed a negativecorrelation with LTA, TNFRSF13B, TMIGD2, KLRC1, TNFSF25, CD40LG, among others. As for immune inhibited genes, FDX1 expression was positively relevant to TGFBR1, PDCD1LG2, IDO1 and IL10RB, while negatively with KIR2DL1, KIR2DL3, PDCD1 and ADORA2A. For chemokine and chemokine receptor genes, FDX1 expression was positively related to CCR2, CCR5, CCR10, CXCL10, CCL2, CXCL9, CXCL11, CCL8 and CXCL6, while negatively related to CXCR1, CXCR3, CCR3, CCR6, CCL21, CXCL17, CCL28 and CCL14. Finally, we explored the relevance between FDX1 expression and anti-gene presentation related genes, the results revealed that almost all of the genes were positively associated with FDX1 expression (Fig. 9B). Consequently, our findings suggest that FDX1 may serve as a cancer immune checkpoint in glioblastoma. Moreover, these results provide a foundation for further research on the anti-tumor activity and immune checkpoint effects of FDX1 in GBM.

### Exploring the therapeutic response in the high and low risk groups

**Fig. 10:**
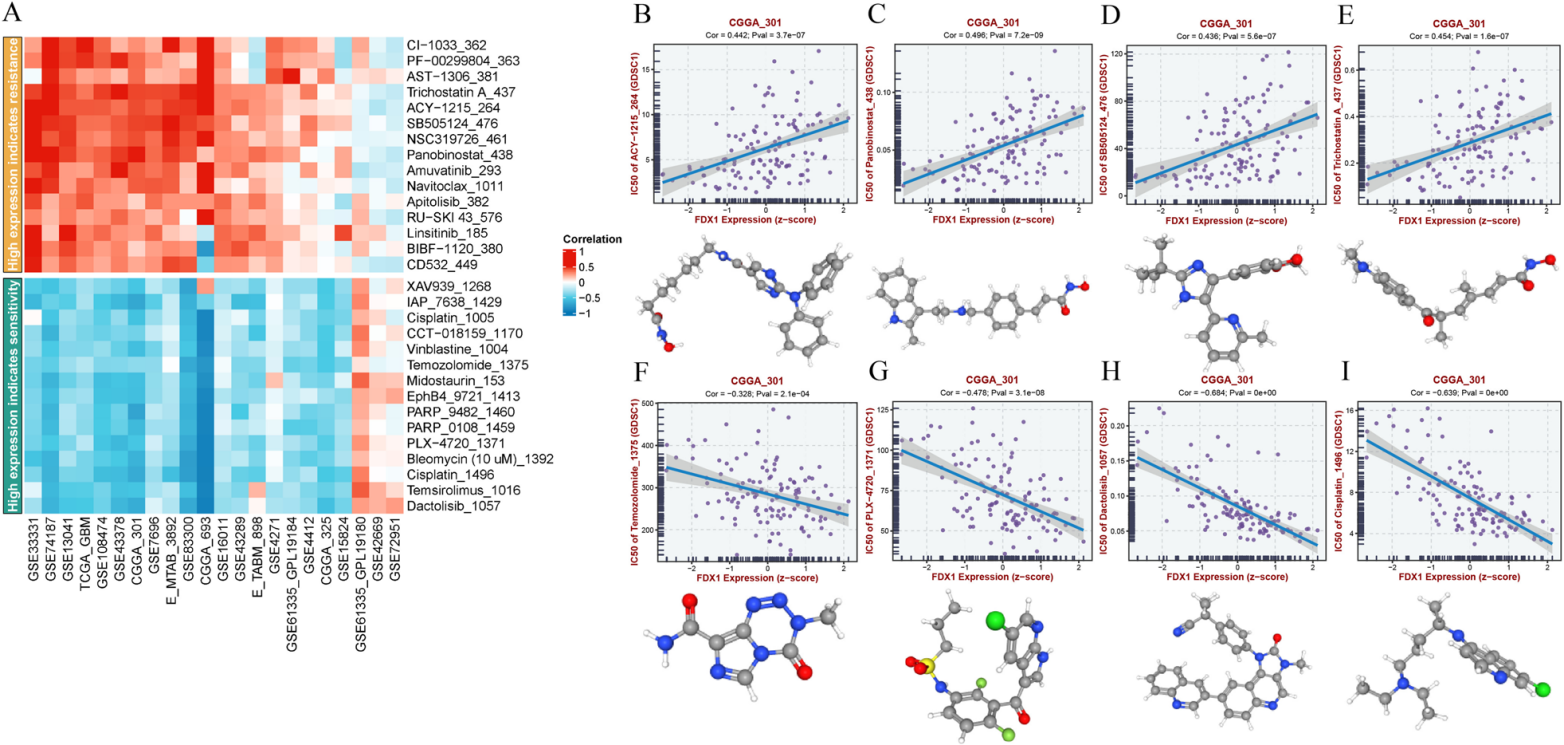
The screened drugs for GBM treatment. **A** The top 15 drugs that high FDX1 expression indicates sensitivity and resistance. **B-E** IC 50 value of 4 representative drugs that indicate resistance. **F-I** IC 50 value of 4 representative drugs that indicate sensitivity

In order to predict the impact of FDX1 expression on chemotherapy, we utilized the pRRophetic algorithm to evaluate the response to chemotherapy based on the half-maximal inhibitory concentration (IC50) for patients with glioblastoma multiforme (GBM) in the GDSC database. Our analysis revealed that there were 15 drugs that exhibited a sensitive response to high FDX1 expression and 15 drugs that demonstrated resistance to high FDX1 expression (Fig. 10A). Subsequently, we identified four drugs from the CGGA database that exhibited the highest correlation within each of these two groups, based on a comprehensive analysis of the TCGA, CGGA, and GEO databases.High risk group was most resistant to ACY-1215 (*P* = 3.7e-07, Fig. 10B), Panobinostat (*P* = 7.2e-09, Fig. 10C), SB505124 (*P* = 5.6e-07, Fig. 10D), Trichostatin A (*P* = 1.6e-07, Fig. 10E). but was most sensitive to Temozolomide (P = 2.1e-04, Fig. 10F), PLX-4720 (*P* = 3.1e-08, Fig. 10G), Dactolisib (*P* < 0e -10, Fig. 10H) and Cisplatin (*P* < 0e -10, Fig. 10I). Finally, the above molecular compounds were visualized through PubChem website. Given these findings, we found that some drugs, such as temozolomide [28], are already first-line drugs for GBM patients, while some are still in clinical trials, so our analysis provides potential molecular chemotherapy agents for GBM patients.

### FDX1 participate in regulating GBM cell proliferation, migration and invasion

**Fig. 11:**
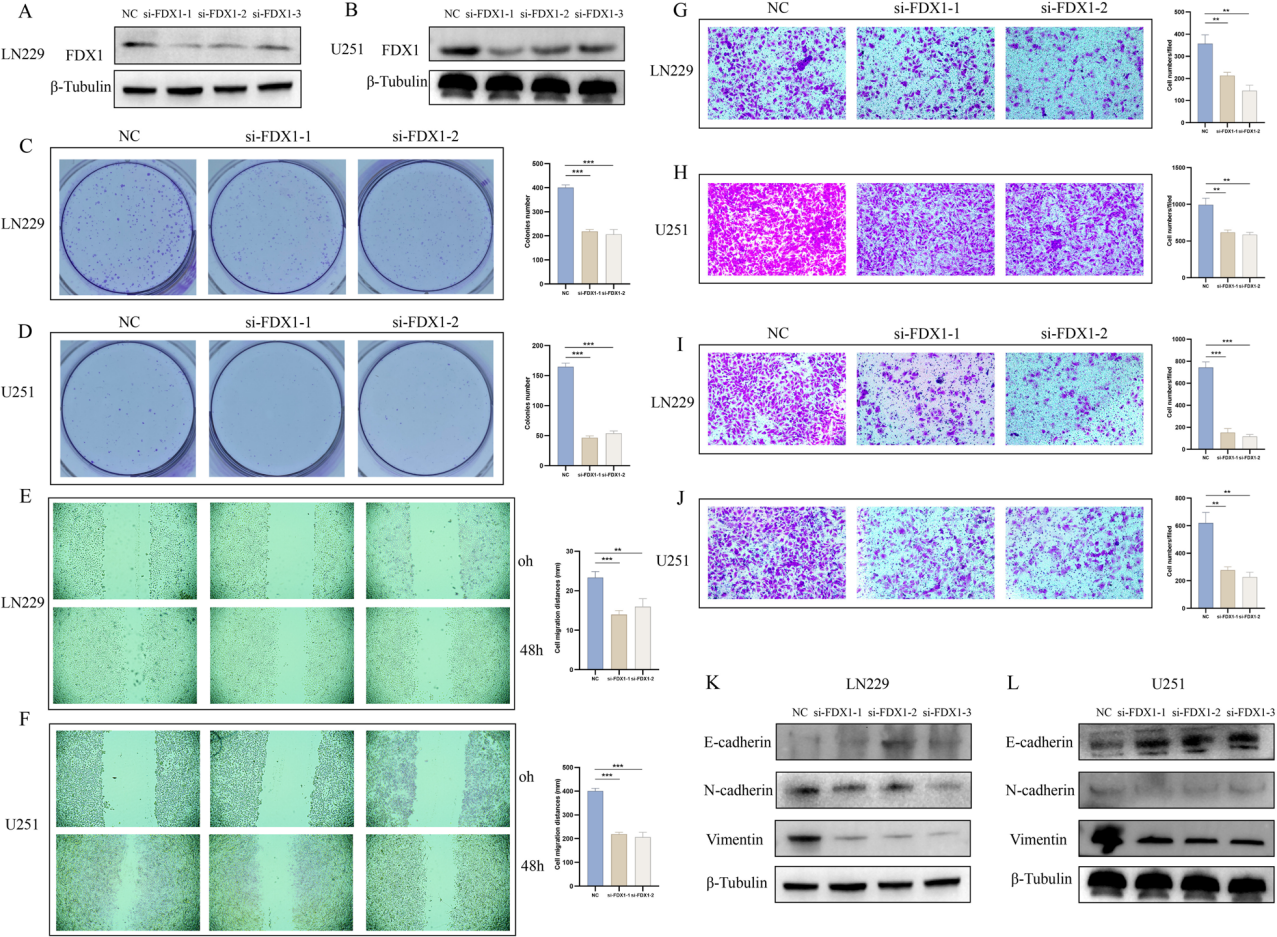
The biological function of FDX1 in GBM cell line was explored in vitro. **A-B** Western blot reveals the knockdown efficiency of two GBM cell lines (LN229 and U251). **C-D** Colony formation assay was performed to explore whether FDX1 can promote cell proliferation. **E–F** Wound healing assay was performed to explore whether FDX1 can promote cell migration. **G-J** Transwell assays were used to explore whether FDX1 can promote cell migration and invasion. **K-L** Western blot was used to explore whether FDX1 can promote epithelial mesenchymal transformation of GBM cells. As for western blot, to hybridize each marker separately, blots were cut based on the molecular weight of the proteins prior to hybridization with primary antibodies

In order to investigate the role of FDX1 in GBM, we opted to conduct additional experiments using the LN229 and U251 cell lines. Initially, transfection with siRNA-FDX1 was performed, followed by the utilization of western blot analysis to assess the efficacy of knockdown (Fig. 11A-B). Since FDX1 is involved in regulating cell proliferation in many other tumors [29], we knocked down the FDX1 expression of LN229 and U251 cells, and then placed the cells in 6-well plates for colony formation experiment, and observed the cell proliferation in different groups 14 days later. The findings demonstrated a significant decrease in cell proliferation in the FDX1 knockdown group, as evidenced by the results presented in Fig. 11C-D. Moreover, the wound healing and Transwell assays revealed a notable reduction in the migration and invasion capabilities of LN229 and U251 cells upon FDX1 knockdown, as depicted in Fig. 11D-J.

Based on previous research findings indicating a significant correlation between FDX1 expression and epithelial-mesenchymal transition (EMT), as well as the ability of FDX1 to promote invasion and migration of GBM cells, this study employed western blot experiments to investigate the impact of FDX1 knockdown on the expressions of E-cadherin, N-cadherin, and Vimentin. The obtained results demonstrated an increase in E-cadherin expression, while N-cadherin and Vimentin expressions decreased (Fig. 11K-L). In conclusion, it can be inferred that FDX1 has the capability to induce the proliferation, migration, and invasion of GBM cell lines. Moreover, it is plausible that FDX1 may facilitate the invasion and migration of GBM cells by activating the EMT pathway.

## Discussion

Glioblastoma, a primary brain tumor characterized by high malignancy, presents a dismal prognosis despite surgical intervention and chemo-radiotherapy, with limited prospects for survival [30]. The growing body of research on programmed cell death has revealed that prognostic models focused on cell death can effectively anticipate the onset and progression of cancer. Additionally, numerous genes associated with cell death have been identified as influential in the regulation of tumor advancement [31, 32]. In March 2022, Todd R Golub's team first discovered that the regulatory mechanism of copper accumulation can contribute to cell death, and they named it “Cuproptosis” [4]. Cuproptosis and cuproptosis-related genes play vital roles in the progression of tumors [5]. For instance, High expression of SLC31A1 is correlated with poor prognosis and dysregulated immune cell infiltration in breast cancer [33]. Cuproptosis-related FDX1 expression and modification levels vary in renal carcinoma, which is associated with tumor cells’ function, immune regulation and prognosis [34]. Cuproptosis-related LIPT1 may facilitate the proliferation and invasion of hepatocellular carcinoma cells, and may be a novel biomarker for hepatocellular carcinoma treatment [25]. Many researchers have also built prognostic models on account of cuproptosis-related genes to predict the survival and prognosis of patients with tumors, for instance, colorectal cancer [35], breast cancer [36] and melanoma [37]. Numerous studies have consistently documented the significant regulatory functions of cuproptosis-related genes in GBM. For example, aberrantly elevated expression of SLC31A1 in GBM have been observed, wherein its heightened levels have been found to facilitate the proliferation and migration of GBM cells while impeding their apoptosis. [38, 39]. Hence, the objective of this study is to develop a prognostic model encompassing cuproptosis-related genes to facilitate the diagnosis and prediction of survival and prognosis in patients with GBM. Additionally, we have specifically chosen the FDX1 gene, known for its significant prognostic impact in GBM, for further investigation. This includes an examination of FDX1 expression, its biological functions, alterations in the tumor immune microenvironment, and its correlation with drug sensitivity in GBM.

We established a prognostic risk model for GBM using 18 (PDHA1, GLS, ATP7B, PDHB, LIAS, GCSH, SLC31A1, FDX1, ATP7A, NLRP3, DBT, MTF1, DLAT, DLD, DLST, NFE2L2, LIPT1 and CDKN2A) cuproptosis-related genes by Cox and Lasso regression analysis. As mentioned above, FDX1 can mediate lipid acylation of proteins to regulate cuproptosis [4], researches have discovered that FDX1 is also relevant to the regulation of multiple tumors, such as FDX1 can promote the cell viability of bladder cancer and prostate cancer [40]. The genes LIPT1, PDHA1, and PDHB are responsible for encoding the lipoic acid pathway, which has been found to have significant implications in tumor progression. Notably, heightened expression of LIPT1 in hepatocellular carcinoma cells has been observed to promote tumor cell proliferation, invasion, and migration, thus highlighting its potential as a viable therapeutic target [25]. Thioctyl has the ability to target DLAT, PDHA1, and PDHB, which in turn can modulate tumor progression by influencing diverse metabolic pathways. Notably, PDHA1 serves as a constituent of the pyruvate dehydrogenase complex and plays a pivotal role in pyruvate metabolism. Suppression of PDHA1 expression leads to a reduction in pyruvate dehydrogenase complex activity, consequently facilitating tumor glycolysis and promoting gastric cancer growth. Conversely, overexpression of PDHA1 and MPC1, along with PG-α, can counteract the Warburg effect and stimulate the proliferation of bile duct cancer cells [41]. Protein fatty acylation, a well-preserved post-translational modification of lysine, has been observed on four enzymes, namely DBT, GCSH, DLST, and DLAT. Additionally, it has been discovered that the suppression of FDX1 or the aforementioned four genes can confer cellular protection against copper toxicity. These significant findings have further stimulated investigations into the regulatory role of FDX1 in protein fatty acylation [4]. Copper homeostasis is primarily regulated by three copper transporters: SLC31A1, ATP7A, and ATP7B. SLC31A1 facilitates copper intake, while ATP7A and ATP7B are responsible for copper transfer. Disruption of copper homeostasis can result in cellular demise [4]. Notably, increased expression of SLC31A1 in GBM has been associated with reduced patient survival, whereas downregulation of SLC31A1 can impede the proliferation, migration, and invasion of GBM cells, thereby promoting a tumor-suppressive microenvironment [38]. Given the significant involvement of FDX1 in cuproptosis, we have subsequently chosen to conduct a more comprehensive investigation on FDX1.

Previous research has demonstrated a significant correlation between cuproptosis and metabolic pathways in various types of cancer. For instance, Zhang Z et al. conducted a non-targeted metabolomics analysis on WT-FDX1 lung adenocarcinoma cells and KD-FDX1 lung adenocarcinoma cells. The findings revealed that the knockdown of the FDX1 gene resulted in a significant increase in fructose 6-phosphate, a key component of glucose metabolism. Additionally, there was a notable increase in acylcarnitine and L-palmitoyl carnitine, both of which are associated with fatty acid metabolism. Furthermore, changes were observed in L-cysteine and L-glutamine metabolites, which are involved in amino acid metabolism [42]. In addition, Ding L et al. discovered that the high FDX1 expression in hepatocellular carcinoma patients is markedly relevant to metabolic, glycolysis and TCA cycle pathways [43]. In this study, it was observed that elevated FDX1 expression in GBM exhibited a positive correlation with various metabolic pathways including amino sugar and nucleotide sugar metabolism, propanoate metabolism, lipoic acid metabolism, riboflavin metabolism, nicotinate and nicotinamide metabolism, and phenylalanine metabolism. Conversely, a negative association was found between FDX1 expression and inositol phosphate metabolism as well as taurine and hypo-taurine metabolism. These findings suggest that FDX1 plays a significant role in glucose metabolism, fatty acid oxidation, and amino acid metabolism within tumor cells, aligning with previous research conclusions.

Due to the profoundly immunosuppressive milieu of glioblastoma and the presence of diverse therapeutic resistance mechanisms, including pronounced tumor heterogeneity, low mutation burden, and localized immune dysfunction, patients with glioblastoma (GBM) experience restricted efficacy from immune checkpoint inhibitors and vaccine therapy [44]. Consequently, ongoing investigations are exploring combination therapies and novel therapeutic approaches alongside immune checkpoint therapy and vaccine therapy. More and more studies have found that tumor-associated macrophages (TAM) can promote tumor immune escape [45, 46], and patients with low T cell infiltration are more likely to deteriorate [47]. Our investigation has revealed a positive correlation between elevated FDX1 expression in glioblastoma multiforme (GBM) patients, increased levels of tumor-associated macrophages, and reduced expression of CD4 + and CD8 + T cells, aligning with previous findings.

Moreover, we conducted a screening of numerous small molecule compounds exhibiting high FDX1 expression, which demonstrated varying levels of sensitivity and resistance among glioblastoma multiforme (GBM) patients. Notably, GBM patients classified within the high-risk group exhibited pronounced resistance to ACY-1215, Panobinostat, SB505124, and Trichostatin A, while displaying heightened sensitivity to Temozolomide, PLX-4720, Dactolisib, and Cisplatin. Temozolomide, being the most commonly employed chemotherapy drug for GBM patients, unfortunately encounters drug resistance in over half of the patient population. Consequently, there is a growing trend towards combining temozolomide with immunotherapy or targeted therapy to investigate the prognosis of GBM patients [28]. For instance, PDIA3P1 promoted the proneural-to-mesenchymal transition by inhibiting C/EBP-β degradation, thus increased the resistance of GBM to temozolomide [48]. High NRF2 expression makes temozolomide-resistant GBM cells sensitive to ferroptosis by up-regulating ABCC1/MRP1 [49]. These outcomes suggest that the potential drugs under consideration may provide novel perspectives for the management of GBM patients, particularly those exhibiting high FDX1 expression. Their combination with small molecule drugs could enhance patient survival and prognosis, in addition to primary therapy.

Simultaneously, our study has revealed that elevated FDX1 expression in GBM cells exerts a stimulatory influence on cell proliferation, invasion, and migration, while GBM cells facilitate invasion via the epithelial mesenchymal transition pathway. In subsequent investigations, we intend to delve deeper into the underlying mechanism through which FDX1 promotes cancer in GBM, as well as explore potential therapeutic avenues.

## Conclusions

In this study, we constructed and validated a prognostic model and nomogram system based on cuproptosis-related genes. These genes have the potential to serve as biomarkers for predicting survival and prognosis in patients with glioblastoma (GBM). Additionally, we focused on investigating FDX1 as the primary subject of our investigation, examining its expression, prognosis, tumor immune microenvironment, substance metabolism pathway, immune cell infiltration, immunotherapy and chemotherapy drug sensitivity in GBM. Furthermore, we conducted in vitro experiments to confirm its biological function.This study has the potential to generate novel prognostic prediction concepts and propose potential therapeutic avenues.

### Supplementary Information

**Additional file 1**

**Additional file 2**

**Additional file 3**

**Additional file 4**

**Additional file 5.**

**Additional file 6.**

**Additional file 7.**

**Additional file 8.**

**Additional file 9.**

**Additional file 10.**

**Additional file 11.**

**Additional file 12.**

**Additional file 13.**

**Additional file 14.**

**Additional file 15.**

**Additional file 16.**

**Additional file 17.**

**Additional file 18.**

**Additional file 19.**

**Additional file 20.**

**Additional file 21.**

**Additional file 22.**

**Additional file 23.**

## Data Availability

All data are available on public repositories, which are listed in the main context. All data included in this study are available by contacting the corresponding authors.
