# Supplementary figures and images for: Systematic analysis based on the cuproptosis-related genes identifies ferredoxin 1 as an immune regulator and therapeutic target for glioblastoma
Source: BMC Cancer. 2023 Dec 19;23:1249. doi: 10.1186/s12885-023-11727-z (PMC10731758; doi:10.1186/s12885-023-11727-z)

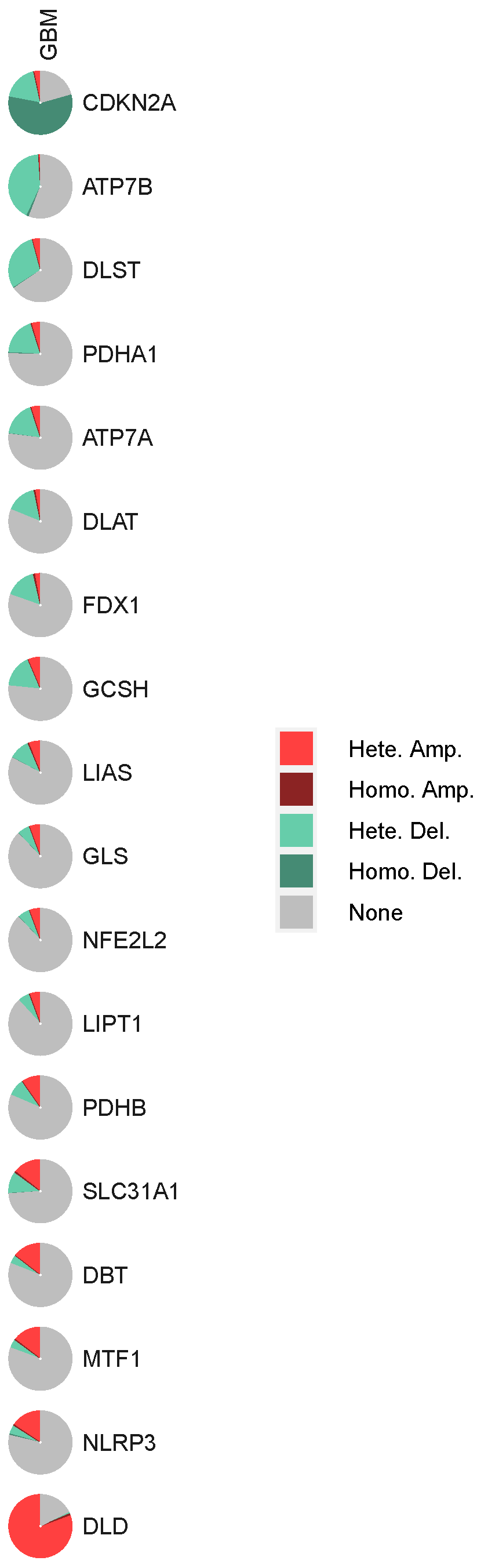

Supplement: Supplementary file 1 — Additional file 1 [file 12885_2023_11727_MOESM1_ESM.tif]

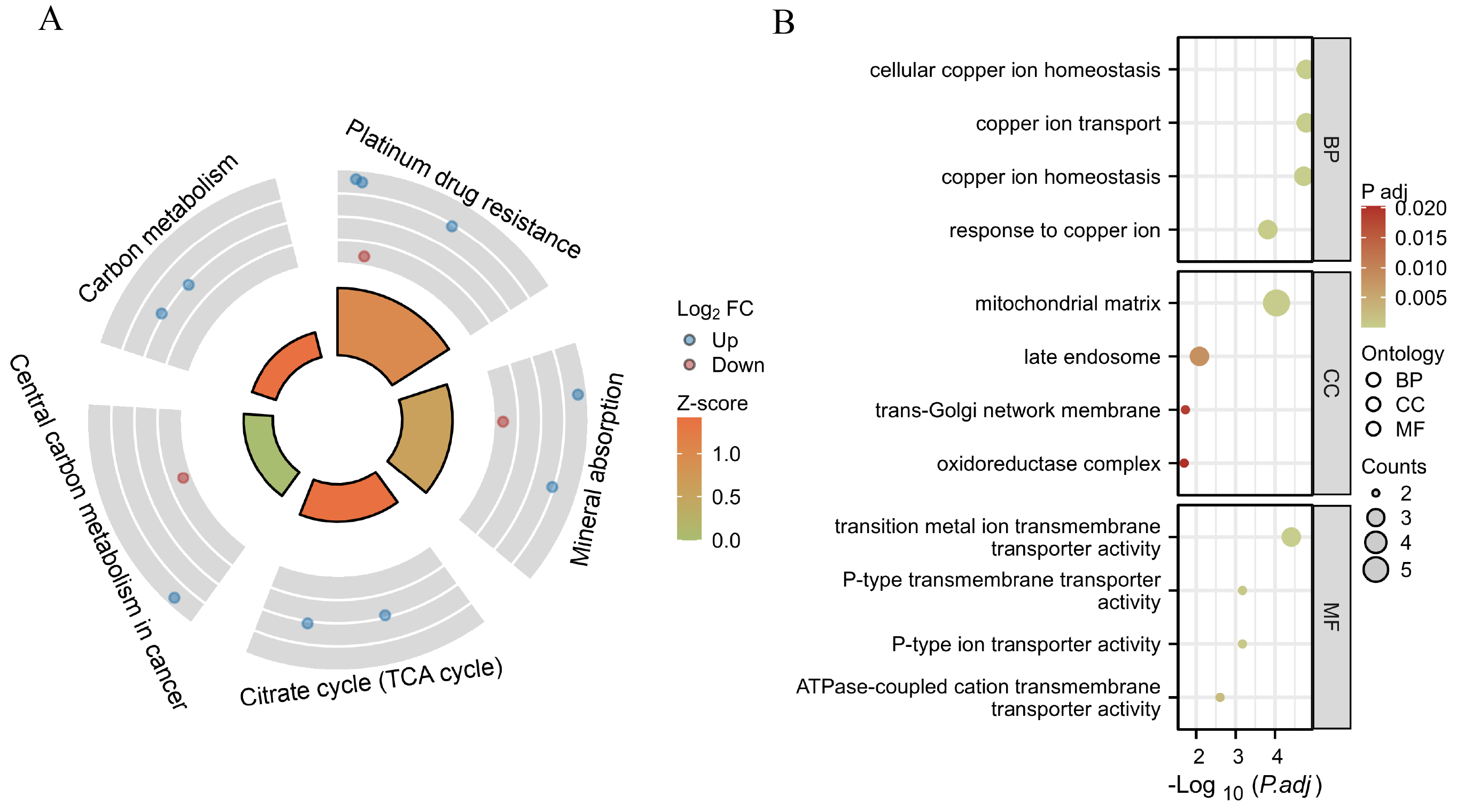

Supplement: Supplementary file 2 — Additional file 2 [file 12885_2023_11727_MOESM2_ESM.tif]

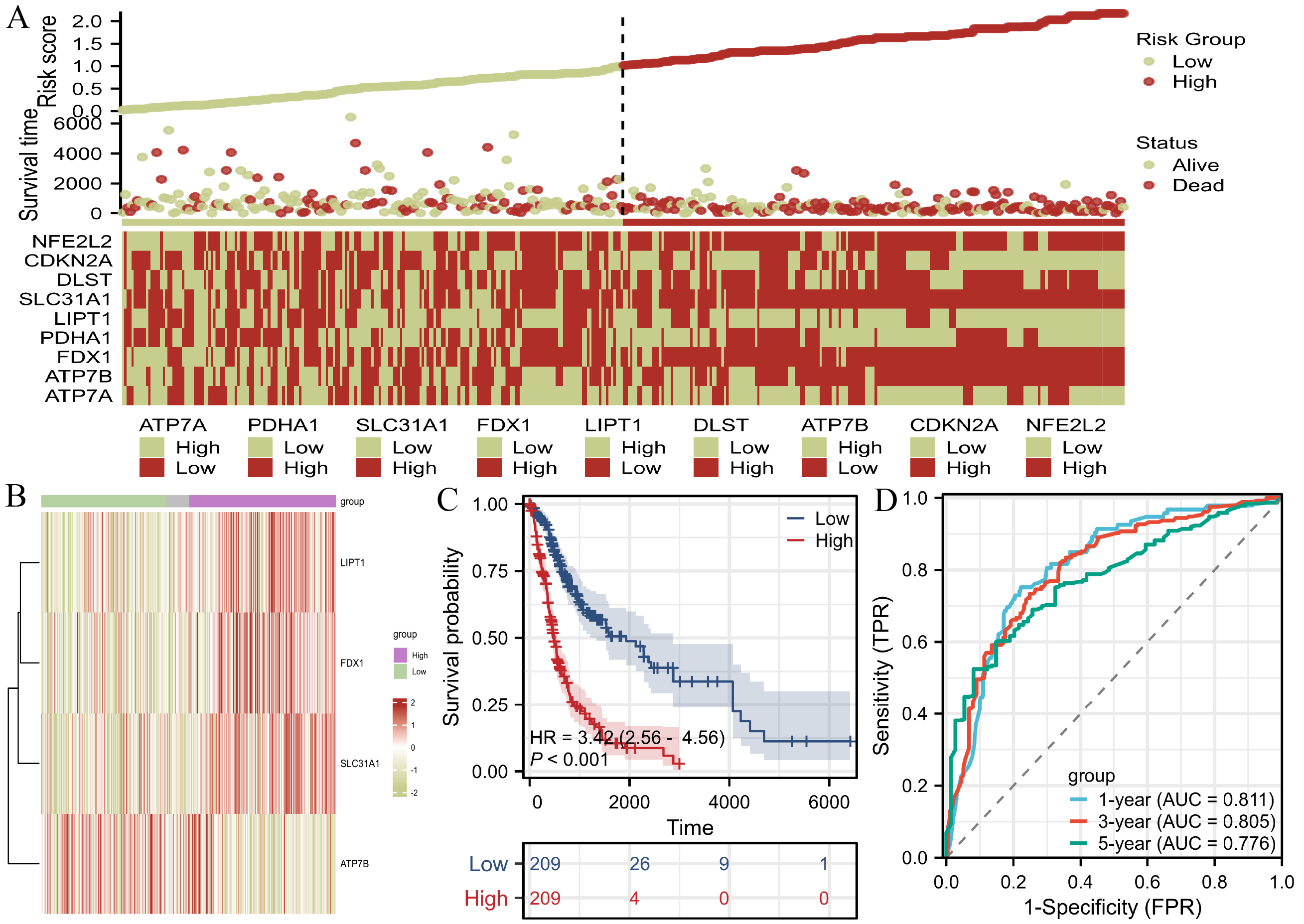

Supplement: Supplementary file 3 — Additional file 3 [file 12885_2023_11727_MOESM3_ESM.tif]

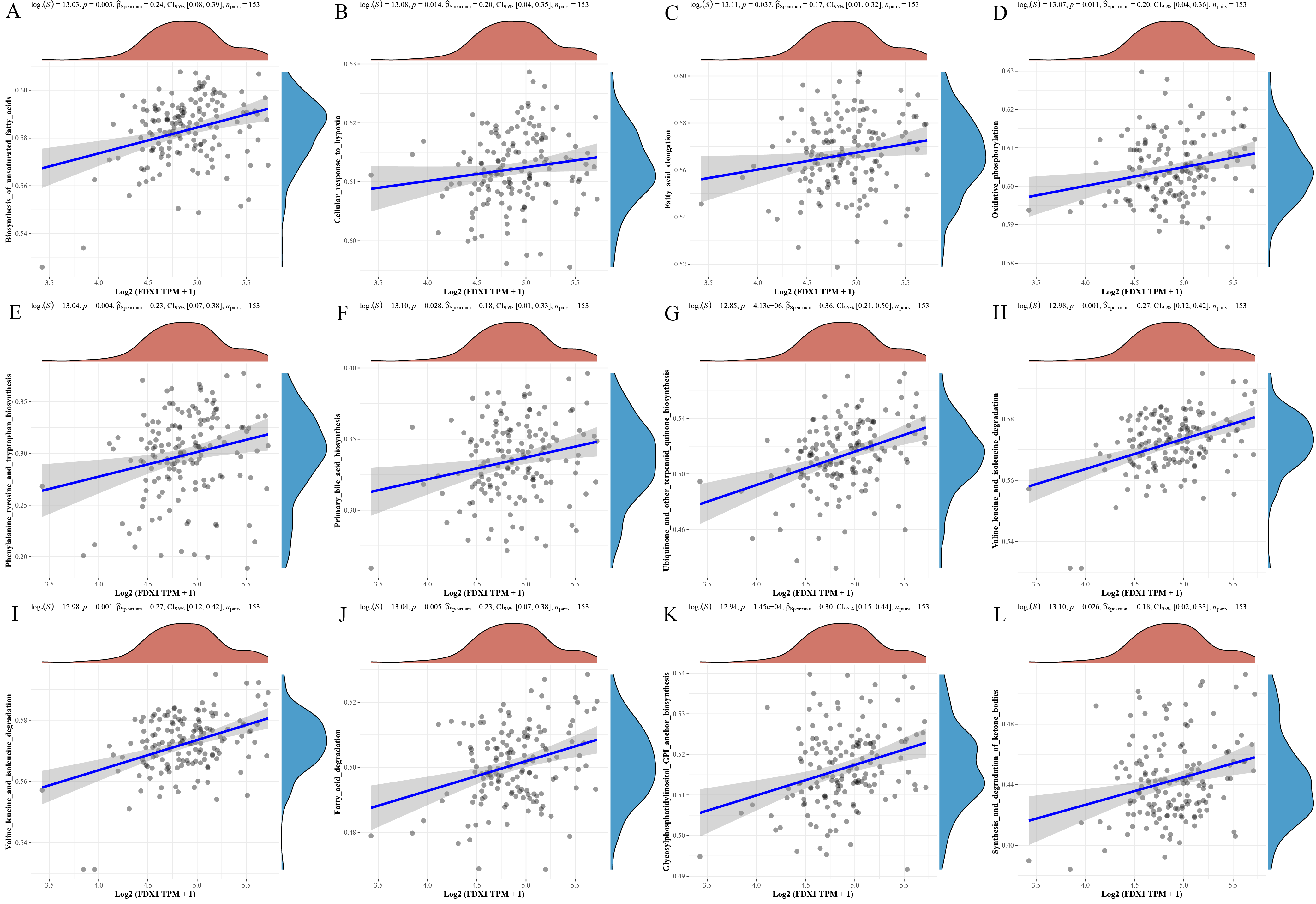

Supplement: Supplementary file 4 — Additional file 4 [file 12885_2023_11727_MOESM4_ESM.tif]
